# Trends in Daily Nicotine Vaping and Unsuccessful Quit Attempts in Youths

**DOI:** 10.1001/jamanetworkopen.2025.41061

**Published:** 2025-11-03

**Authors:** Abbey R. Masonbrink, Dayoung Bae, Junhan Cho, Richard A. Miech, Hongying D. Dai, Alyssa F. Harlow, Steve Sussman, Dae-Hee Han, Louisiana M. Sanchez, Abigail Adjei, Leah R. Meza, Ming Li, Adam M. Leventhal

**Affiliations:** 1 Keck School of Medicine, University of Southern California, Los Angeles; 2 Department of Pediatrics, Children’s Hospital Los Angeles, Los Angeles, California; 3 Institute for Addiction Science, University of Southern California, Los Angeles; 4 Institute for Social Research, University of Michigan, Ann Arbor; 5 College of Public Health, University of Nebraska, Omaha; 6 Rollins School of Public Health, Emory University, Atlanta, Georgia

## Abstract

**Importance:**

US youths who vape nicotine may be hardening into a more nicotine-dependent, treatment-resistant population over time, as evidenced by shifts toward daily vaping and difficulty quitting, which may vary by behavioral health and demographic factors.

**Objective:**

To assess prevalence trends from 2020 to 2024 in (1) current (past 30 days) nicotine vaping, (2) daily vaping among youths who currently vape nicotine, and (3) unsuccessful quit attempts among youths who vape nicotine daily and to examine variation in these trends by behavioral health and demographic factors.

**Design, Setting, and Participants:**

This cross-sectional study used data from Monitoring the Future (MTF), a nationally representative annual cross-sectional survey of 8th, 10th, and 12th grade respondents in US middle and high schools, from survey years 2020 to 2024.

**Exposures:**

Survey year; grade in school; population density of school location; and self-reported sex, race and ethnicity, depressive symptoms, conduct problems, and past-30-day use of nicotine vaping, other tobacco products, cannabis, and alcohol.

**Main Outcomes and Measures:**

Prevalence of (1) past-30-day nicotine vaping (≥1 vs 0 days) among all respondents, (2) past-30-day daily vaping among currently vaping youths (vaped all 30 vs 1-29 days), and (3) unsuccessful attempts to quit vaping (yes, no) among youths vaping daily, with weights applied to generate nationally representative estimates.

**Results:**

In the pooled sample of 115 191 MTF respondents (50.8% [95% CI, 50.0%-51.6%] male), prevalence of past-30-day nicotine vaping declined from 2020 to 2024 (risk ratio [RR], 0.88; 95% CI, 0.86-0.89). Among youths who currently vaped (n = 15 226), prevalence of daily vaping rose from 15.4% (95% CI, 13.1%-18.0%) in 2020 to 28.8% (95% CI, 26.6%-31.0%) in 2024 (RR, 1.14; 95% CI, 1.11-1.18). Among daily vapers (n = 3512), prevalence of unsuccessful quit attempts increased from 28.2% (95% CI, 19.5%-38.8%) in 2020 to 53.0% (95% CI, 45.9%-60.0%) in 2024 (RR, 1.08; 95% CI, 1.02-1.15). For female, non-Hispanic Black, cannabis-using, and tobacco-using youths, past-30-day nicotine vaping prevalence either remained stable or reduced more slowly during 2020 to 2024 compared with the overall study population (eg, from 10.4% [95% CI, 8.6%-12.5%] to 4.1% [95% CI, 3.5%-4.8%] in those without vs 58.1% [95% CI, 51.9%-64.1%] to 57.3% [95% CI, 54.0%-60.5%] in those with past-30-day cannabis use). Among current vapers, daily vaping increased more rapidly in rural youths (from 16.4% [95% CI, 11.5%-22.9%] to 41.8% [95% CI, 35.3%-48.5%]) than in urban youths (15.9% [95% CI, 12.7%-19.6%] to 18.1% [95% CI, 14.8%-21.9%]).

**Conclusions and Relevance:**

The findings of this cross-sectional study of US youths suggest that although the prevalence of current nicotine vaping declined during 2020 to 2024, the youth vaping population may have hardened over this period, evidenced by increasing daily use, more unsuccessful quit attempts, and shifting demographic profiles. Clinicians and policy makers should be mindful that youths with frequent vaping increasingly face unique challenges that may impact treatment and prevention efforts.

## Key Points

**Question:**

Did nicotine vaping patterns of US youths change from 2020 to 2024?

**Findings:**

In this cross-sectional study of 115 191 youths in 8th to 12th grade, weighted prevalence of daily nicotine vaping rose from 15% in 2020 to 29% in 2024 among current vapers and unsuccessful quit attempts increased from 28% to 53% among daily vapers.

**Meaning:**

The findings suggest that the US youth nicotine vaping population recently became increasingly represented by daily use and unsuccessful quit attempts, a trend of which clinicians and policy makers should be aware.

## Teaser

This cross-sectional study examines trends in rates of nicotine vaping and unsuccessful quit attempts from 2020 to 2024 among US youths in 8th, 10th, and 12th grade.

## Introduction

Despite recent declines in youth nicotine vaping prevalence, an estimated 1.6 million US youths reported current (past 30 days) nicotine vaping in 2024.^1^ As the youth vaping population shrinks, concern remains that those who continue to vape are increasingly displaying daily use patterns and behavioral health comorbidities, indicating possible hardening.^1^ Hardening was originally framed in the adult smoking cessation literature as the concept that as the smoking prevalence in the overall population declines, the remaining users represent a more nicotine-dependent, treatment-resistant population.^2^ Evidence describing whether youths who vape nicotine are hardening over time and identifying groups at elevated risk for hardening could inform future prevention and treatment programs and policies.

A previous study found that the number of nicotine vaping days per month and use of nicotine vaping within 5 minutes of waking increased among US youths between 2014 and 2021, suggestive of hardening.^1^ However, this study evaluated the previous decade, with unknown recent generalizability; did not focus on characterizing daily users; and did not evaluate trends in unsuccessful cessation attempts among daily vapers—a key feature of hardening. One reason to examine recent trends of hardening in the youth vaping population is that e-cigarette markets have evolved toward products with higher nicotine potency,^3,4^ which may carry heightened risk for nicotine dependence and withdrawal symptoms.^1,5^ Nicotine use is associated with numerous adverse health outcomes including potential cognitive implications (such as attention, learning, and memory problems), worsening of emotional disorders, and cardiovascular issues and is also associated with co-use of other substances including tobacco products, cannabis, and alcohol.^5,6,7,8,9,10,11,12,13,14^ Because of the recent shifts toward products with higher nicotine concentration,^15^ and as vaping has become less common and socially normative in youths,^16^ it is reasonable to hypothesize that the youth vaping population might be increasingly represented by daily, relapsing use patterns and by individuals with certain demographic and related behavioral health risk factors over recent years.

This study investigated whether the US youth nicotine vaping population hardened from 2020 to 2024 by estimating cross-year trends in 3 nested outcomes: (1) prevalence of current vaping (ie, past 30 days) in the overall population, (2) proportion of currently vaping youths who vape nicotine daily, and (3) proportion of daily vaping youths who report a history of unsuccessful quit attempts—a cardinal symptom of nicotine dependence.^1,17^ Secondarily, this study examined whether youths with behavioral health problems, polysubstance use, or demographic risk factors displayed differential trends (ie, differences in the direction or shape of the slope) in these outcomes between characteristic groups and subgroups.

## Methods

### Data Source

In this cross-sectional study, we analyzed 5 years of cross-sectional data collected by Monitoring the Future (MTF), an annual in-school web-based survey that is nationally representative of students in 8th, 10th, and 12th grades.^18^ This study was based in the socioecological framework, which emphasizes the role of multilevel factors in behaviors, including individual-level factors (eg, demographics, mental health), social and behavioral factors (eg, polysubstance use), and contextual factors (eg, rurality, grade level). All measures were informed by previous literature on hardening among youths.^1,17^ MTF first assessed daily use of nicotine vaping among current users in 2020; thus, we used the survey years from 2020 through 2024. Due to the COVID-19 pandemic, data collection was halted prematurely in 2020, and some surveys were completed remotely in 2021 and 2022. However, descriptive analyses indicated that the results of the shortened 2020 MTF data collection did not differ from the nationally representative results of previous years.^17^ Sampling, measures, and other survey methods remained unchanged from 2020 to 2024. This study was approved by the University of Michigan institutional review board (IRB). Informed consent (active [ie, written consent provided] or passive [ie, consent not actively revoked in writing], per school policy) to participate in the MTF was obtained from parents for students aged younger than 18 years and from students if they were aged 18 years or older. Consent for this current analysis was waived by the University of Michigan IRB because we used deidentified MTF data. This study followed the Strengthening the Reporting of Observational Studies in Epidemiology (STROBE) reporting guideline.

### Outcome Measures

Three binary outcome measures were assessed annually via self-reported survey items. Current nicotine vaping was assessed among all MTF respondents (≥1 vs 0 days within the past 30 days). Among those who reported current use, daily vaping in the past 30 days was assessed (all 30 days vs ≤29 days). Among daily nicotine vapers, unsuccessful quitting was assessed by asking whether they had ever tried to stop vaping nicotine but were unable to (yes vs no).

### Exposure Measures

Demographic variables included grade (8th, 10th, or 12th); self-reported sex (female, male); self-reported race and ethnicity (Hispanic or Latino [included Cuban American, Mexican American or Chicano, Puerto Rican, or Other Hispanic or Latino], non-Hispanic Black, non-Hispanic White, or another race and ethnicity [included American Indian or Alaska Native, Asian American, Middle Eastern, or Native Hawaiian or Other Pacific Islander]), assessed to describe the study sample; and population density based on school location (ie, urban [large standard metropolitan statistical area (SMSA)], suburban or town [other SMSA], or rural [non-SMSA]). Conduct problems were assessed using 5 self-reported items administered to a randomly selected subsample, with individuals reporting 1 or more conduct behaviors (such as “hurt someone badly” or “taken something not belonging to you worth over $50”) on 1 or more occasions within the past 12 months classified as having a conduct problem. Depressive symptoms, administered to a randomly selected subsample, were measured by summing four 5-point Likert scale items^17,18,19^ (eg, “life often seems meaningless,” “the future often seems hopeless”) for a total score ranging from 4 to 20 (with higher scores reflecting more reported depressive symptoms), which was then divided by the median in the overall MTF sample. The total depressive symptom score was dichotomized at the sample median (≥9 indicating depressive symptoms and ≤8 indicating no depressive symptoms). To assess polysubstance use, survey items included past-30-day use of other tobacco products (defined as using ≥1 vs 0 of the following: cigarettes, smokeless tobacco, large cigars, flavored little cigars [cigarillos], regular (unflavored) cigarillos, and hookah tobacco), cannabis (yes, no), or alcohol (yes, no).

### Statistical Analysis

Three nested analytic samples were created for the 3 respective outcomes (past-30-day nicotine vaping, past-30-day daily nicotine vaping, and unsuccessful quitting), each of which pooled data across years. After reporting descriptive results of respondent characteristics within each analytic sample, we calculated weighted proportions of students with each outcome by study year. We used log-binomial regression to model each outcome as a function of survey year (2020-2024), incorporating both linear (ie, slope over time) and quadratic curvilinear (ie, curvature of slope; year × year) terms. To explore differences in cross-year trends in vaping outcomes as a function of respondent characteristics, survey year × characteristic interactions were tested using omnibus Wald tests. Significant interactions were followed by post hoc analyses of cross-year linear and quadratic trends as well as proportion changes from 2020 to 2024 estimates, stratified by characteristics. Risk ratios (RRs) and corresponding 95% CIs were reported with multiple-testing significance corrections using the Benjamini-Hochberg procedure to control the study-wise false discovery rate at 0.05 (2-tailed).^19^ Missing data were addressed via listwise deletion. Analyses were conducted using Stata, version 18.5 (StataCorp LLC). We applied weights to generate nationally representative estimates, with SEs adjusted for clustering within schools and regional sampling strata. Two-sided *P* < .05 was considered significant.

## Results

### Sample Characteristics

The overall analytic sample comprised all 115 191 MTF participants with complete data (missing data ranged from 2.8% to 6.8% per variable) (eFigure in Supplement 1), of whom 33.8% (95% CI, 29.3%-38.7%) were 8th graders, 35.1% (95% CI, 30.4%-40.0%) were 10th graders, and 31.1% (95% CI, 95% CI, 26.7%-35.9%) were 12th graders. Among the respondents, 49.2% (95% CI, 48.4%-50.0%) were female and 50.8% (95% CI, 50.0%-51.6%) were male; 21.1% (95% CI, 18.7%-23.8%) self-identified as Hispanic or Latino, 12.5% (95% CI, 10.9%-14.1%) as non-Hispanic Black, 46.2% (95% CI, 43.4%-49.0%) as non-Hispanic White, and 20.2% (95% CI, 19.0%-21.4%) as another race and ethnicity (Table 1). The analytic subsamples limited to those with past-30-day vaping (n = 15 226) and daily vaping (n = 3512) differed in some characteristics from the overall MTF sample. Most students with daily vaping scored above the median depression score (75.9%; 95% CI, 72.3%-79.2%), reported at least 1 conduct problem (50.5%; 95% CI, 44.6%-56.4%), and reported polysubstance use, especially cannabis (68.8%; 95% CI, 66.6%-70.9%) or alcohol (64.4%; 95% CI, 62.1%-66.7%). Depression items administered to a randomly selected subsample reduced the analytic sample for depressive symptoms to 44 390 for all MTF respondents, 5402 for past-30-day vapers, and 1253 for daily vapers; conduct items administered to a randomly selected subsample reduced the analytic sample for conduct problems to 25 045 for all MTF respondents, 2855 for past-30-day vapers, and 587 for daily vapers.

**Table 1.** Participant Characteristics Within Each Analytic Sample, 2020-2024 Pooled

Characteristic	Participants, weighted % (95% CI)		
	All MTF respondents (N = 115 191)	Past-30-d vaping (n = 15 226)	Daily vaping (n = 3512)^a^
Grade			
8	33.8 (29.3-38.7)	19.2 (15.2-24.0)	11.7 (8.7-15.6)
10	35.1 (30.4-40.0)	35.9 (30.3-41.9)	31.4 (26.1-37.3)
12	31.1 (26.7-35.9)	44.9 (38.8-51.1)	56.9 (50.4-63.1)
Sex			
Female	49.2 (48.4-50.0)	54.3 (52.4-56.2)	53.2 (50.8-55.6)
Male	50.8 (50.0-51.6)	45.7 (43.8-47.6)	46.8 (44.3-49.2)
Race and ethnicity			
Hispanic or Latino^b^	21.1 (18.7-23.8)	16.3 (13.9-19.1)	11.1 (9.1-13.3)
Non-Hispanic Black	12.5 (10.9-14.1)	8.2 (7.1-9.4)	5.8 (4.7-7.2)
Non-Hispanic White	46.2 (43.4-49.0)	55.7 (52.4-58.9)	62.6 (58.9-66.2)
Another race and ethnicity^c^	20.2 (19.0-21.4)	19.8 (18.4-21.3)	20.5 (18.2-23.0)
Population density			
Urban	33.6 (29.4-38.0)	24.4 (20.1-29.2)	20.0 (15.8-25.0)
Suburban or town	49.3 (44.9-53.8)	50.9 (45.5-56.2)	49.9 (44.4-55.4)
Rural	17.1 (14.8-19.6)	24.7 (20.8-29.1)	30.0 (25.7-34.8)
≥1 Conduct problem^d^	30.2 (29.4-31.1)	53.2 (50.3-56.1)	50.5 (44.6-56.4)
Depressive symptoms^e^	57.6 (56.9-58.3)	69.1 (67.2-71.0)	75.9 (72.3-79.2)
Substance use in past 30 d			
Tobacco^f^	3.9 (3.7-4.1)	18.9 (17.9-20.0)	26.5 (24.3-28.7)
Cannabis^g^	13.1 (12.8-13.4)	57.7 (56.5-58.9)	68.8 (66.6-70.9)
Alcohol	15.6 (15.3-15.9)	55.0 (53.8-56.2)	64.4 (62.1-66.7)

Abbreviation: MTF, Monitoring the Future.

^a^ Daily vaping was defined as vaping on all 30 days during the past 30-day period.

^b^ Indicates individuals identifying as Mexican American or Chicano, Cuban American, Puerto Rican, or Other Hispanic or Latino.

^c^ Indicates individuals identifying as American Indian or Alaska Native, Asian American, Middle Eastern, or Native Hawaiian or Other Pacific Islander.

^d^ Conduct problems were assessed using 5 self-reported items on the frequency of specific behaviors in the past 12 months (any vs none). These items were administered to a randomly selected subsample, reducing the analytic sample to 25 045 for all MTF respondents, 2855 for past-30-day vapers, and 587 for daily vapers.

^e^ Depressive symptoms were assessed using 4 self-reported, yes-no items summed to create a total score. These items were administered to a randomly selected subsample, reducing the analytic sample to 44 390 for all MTF respondents, 5402 for past-30-day vapers, and 1253 for daily vapers. Participants scoring above the sample median were classified as having depressive symptoms.

^f^ Included cigarettes, smokeless tobacco, large cigars, flavored little cigars (cigarillos), regular (unflavored) cigarillos, or tobacco using a hookah.

^g^ Smoking or vaping marijuana.

### Cross-Year Trends

**Figure 1.**
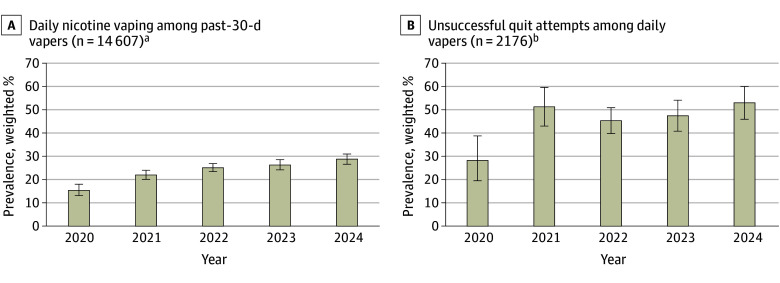
Prevalence of Daily Nicotine Vaping and Unsuccessful Quit Attempts, 2020 to 2024 Whiskers represent 95% CIs. ^a^Significant linear trend (*P* < .001) and quadratic trend (*P* = .002). ^b^Daily vapers reported nicotine vaping on all of the past 30 days, as shown in the eTable in Supplement 1. Significant linear trend (*P* = .01).

Among all MTF respondents, the prevalence of past-30-day nicotine vaping use significantly decreased from 2020 to 2024 (linear RR, 0.88; 95% CI, 0.86-0.89) (Table 2).^20^ Among students with past 30-day nicotine vaping, prevalence of daily use significantly increased from 15.4% (95% CI, 13.1%-18.0%) in 2020 to 28.8% (95% CI, 26.6%-31.0%) in 2024 (linear RR, 1.14; 95% CI, 1.11-1.18) (Figure 1) following a decelerating curvature trend in which increases in later years were smaller than in earlier years (quadratic RR, 0.96; 95% CI, 0.93-0.98). Among youths who vaped daily, the prevalence of unsuccessful quit attempts significantly increased linearly from 28.2% (95% CI, 19.5%-38.8%) in 2020 to 53.0% (95% CI, 45.9%-60.0%) in 2024 (linear RR, 1.08; 95% CI, 1.02-1.15) (Figure 1).

**Table 2.** Prevalence of Daily Nicotine Vaping in Past 30 Days and Unsuccessful Quit Attempt, 2020-2024

Sample group	Participants^a^					Time trend			
	2020	2021	2022	2023	2024	Linear		Quadratic	
						RR (95% CI)	*P* value	RR (95% CI)	*P* value
**Overall **									
Participants, No.	11 484	30 124	29 275	21 282	23 026	NA	NA	NA	NA
Any vaping in past 30 d^b^	17.8 (16.9-18.9)	13.2 (12.7-13.8)	13.8 (13.3-14.3)	11.8 (11.2-12.4)	10.1 (9.6-10.6)	0.88 (0.86-0.89)	<.001^c^	1.01 (0.99-1.02)	.12
**Past-30-d vapers^d^**									
Participants, No.	1484	3937	4011	2786	2389	NA	NA	NA	NA
Daily vaping^e^	15.4 (13.1-18.0)	22.0 (20.1-24.0)	25.1 (23.4-26.9)	26.3 (24.2-28.6)	28.8 (26.6-31.0)	1.14 (1.11-1.18)	<.001^c^	0.96 (0.93-0.98)	.002^c^
**Past-30-d daily vapers^f^**									
Participants, No.	134	539	650	416	437	NA	NA	NA	NA
Unsuccessful quit attempt^g^	28.2 (19.5-38.8)	51.3 (43.0-59.6)	45.3 (39.8-50.9)	47.4 (40.8-54.1)	53.0 (45.9-60.0)	1.08 (1.02-1.15)	.01^c^	0.97 (0.93-1.01)	.19

Abbreviations: NA, not applicable; RR, risk ratio.

^a^ Data are presented as weighted percentage (95% CI) of participants unless otherwise indicated.

^b^ Vaped nicotine at least once in the given time frame.

^c^ Statistically significant after Benjamini-Hochberg correction for multiple tests to maintain a study-wise false discovery rate of .05.

^d^ In 2020, some respondents reported past-30-day nicotine vaping using the “20 or more days” category instead of “all past 30 days,” reducing the analytic sample for daily vaping to 1484 from 2103.

^e^ Defined as vaping on all 30 days during the past-30-day period.

^f^ Represents respondents who both vaped daily in the past 30 days and also provided valid responses to the unsuccessful quit attempt question. The total number of respondents who vaped daily in the past 30 days was 234 in 2020, 817 in 2021, 1028 in 2022, 745 in 2023, and 688 in 2024.

^g^ Defined as reporting ever trying to quit vaping nicotine but being unable to do so.

### Differences in Trends in Nicotine Vaping Outcomes by Student Characteristics

#### Any Vaping in Past 30 Days

**Figure 2.**
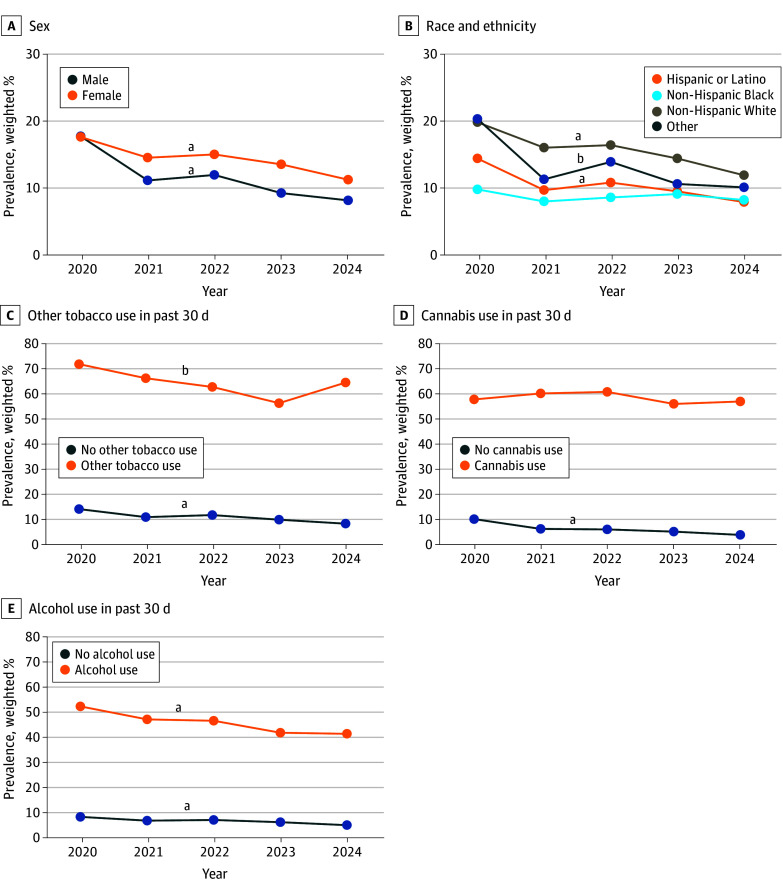
Trends in Past-30-Day Nicotine Vaping Among All Monitoring the Future Respondents, Stratified by Participant Characteristics B, Other race and ethnicity included American Indian or Alaska Native, Asian American, Middle Eastern, or Native Hawaiian or Other Pacific Islander. ^a^Significant linear time trend (*P* < .05). ^b^Significant linear and quadratic time trends (*P* < .05).

For the past-30-day nicotine vaping outcome among all MTF respondents, there were significant 2-way interactions of year with sex (*F*_4_ = 7.15; *P* < .001); with race and ethnicity (*F*_12_ = 2.75; *P* = .001); and with use of tobacco (*F*_4_ = 12.80; *P* < .001), cannabis (*F*_4_ = 46.98; *P* < .001), and alcohol (*F*_4_ = 4.05; *P* = .003) (Figure 2 and eTable in Supplement 1). Past-30-day vaping prevalence declined linearly from 2020 to 2024 in both sexes; however, the rate of decline was steeper in males (from 17.8% [95% CI, 14.9%-21.1%] to 8.2% [95% CI, 7.0%-9.6%], a 53.9% proportional reduction) than in females (from 17.7% [95% CI, 15.2%-20.6%] to 11.3% [95% CI, 10.1%-12.7%], a 36.2% proportional reduction). Past-30-day vaping significantly declined in a linear form from 2020 to 2024 for non-Hispanic White (from 19.8% [95% CI, 17.2%-22.8%] to 11.9% [95% CI, 10.4%-13.7%], a 39.9% proportional reduction) and Hispanic or Latino (from 14.4% [95% CI, 10.5%-19.4%] to 7.9% [95% CI, 6.6%-9.5%], a 45.1% proportional reduction) youths but did not change significantly over time among non-Hispanic Black youths (from 9.8% [95% CI, 5.9%-15.6%] in 2020 to 8.2% [95% CI, 5.9%-15.6%] in 2024, a 16.3% proportional reduction). Among youths without past-30-day use of other tobacco products, past-30-day nicotine vaping significantly linearly declined from 2020 to 2024 (from 14.4% [95% CI, 12.3%-16.8%] to 8.6% [95% CI, 7.6%-9.6%], a 40.3% proportional reduction). By contrast, in youths with past-30-day use of other tobacco products, there was a quadratic U-shaped significant trend in which past-30-day nicotine vaping dipped from 72.0% (95% CI, 66.1%-77.2%) in 2020 to 56.5% (95% CI, 50.5%-62.3%) in 2023 and rebounded to 64.7% (95% CI, 59.4%-69.6%) in 2024, reflecting a 10.1% proportional reduction from 2020 to 2024. Past-30-day nicotine vaping significantly linearly declined from 2020 to 2024 among those without past-30-day cannabis use (from 10.4% [95% CI, 8.6%-12.5%] to 4.1% [95% CI, 3.5%-4.8%], a 60.6% proportional reduction) but did not significantly change across time in participants with past-30-day cannabis use (from 58.1% [95% CI, 51.9%-64.1%] to 57.3% [95% CI, 54.0%-60.5%], a 1.4% proportional reduction). Past-30-day nicotine vaping significantly linearly declined with a steeper slope among youths without past-30-day alcohol use (from 8.7% [95% CI, 7.4%-10.2%] to 5.4% [95% CI, 4.8%-6.2%], a 37.9% proportional reduction) than among those with past-30-day alcohol use (from 52.6% [95% CI, 48.0%-57.2%] to 41.7% [95% CI, 38.0%-45.6%], a 20.7% proportional reduction).

#### Daily Nicotine Vaping and Unsuccessful Quit Attempts

**Figure 3.**
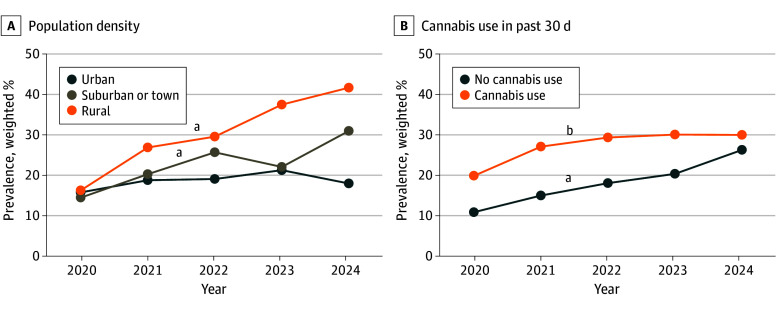
Trends in Past-30-Day Daily Nicotine Vaping Among Past-30-Day Vapers, Stratified by Population Density and Cannabis Use Daily vapers reported vaping on all of the past 30 days. ^a^Significant linear time trend (*P* < .05). ^b^Significant linear and quadratic time trends (*P* < .05).

There were significant interactions of year with population density (*F*_8_ = 3.27; *P* = .001) and past-30-day cannabis use (*F*_4_ = 4.04; *P* = .003) for the daily nicotine vaping outcome (Figure 3 and eTable in Supplement 1). Daily vaping among past-30-day vapers increased linearly from 2020 to 2024 for rural youths (from 16.4% [95% CI, 11.5%-22.9%] to 41.8% [95% CI, 35.3%-48.5%], a 154.9% proportional increase) and suburban youths (from 14.6% [95% CI, 10.5%-20.0%] to 31.1% [95% CI, 27.4%-34.9%], a 113.0% proportional increase) but did not change significantly in urban youths (from 15.9% [95% CI, 12.7%-19.6%] to 18.1% [95% CI, 14.8%-21.9%], a 13.8% proportional increase). Among youths with past-30-day cannabis use, daily nicotine vaping increased linearly (from 20.0% [95% CI, 16.1%-24.6%] to 30.1% [95% CI, 27.3%-33.2%], a 50.5% proportional increase). Among youths without past-30-day cannabis use, a significant quadratic trend in daily nicotine vaping was observed in which the rate of increase accelerated in later years (from 11.0% [95% CI, 8.2%-14.5%] in 2020 to 26.4% [95% CI, 21.0%-32.7%] in 2024, a 140.0% proportional increase). Trends in unsuccessful quit attempts did not differ by respondent characteristics, as evidenced by a lack of significant interactions (eTable in Supplement 1).

## Discussion

Although the percentage of US youths who currently vaped nicotine declined from 2020 to 2024, the youth vaping population may have hardened during this period. Nearly 2-fold prevalence increases were observed for daily vaping among currently vaping youths and for unsuccessful quit attempts among daily vaping youths from 2020 to 2024. Differential cross-year trends in vaping outcomes by respondent characteristics were also observed, indicating that the composition of current vapers or daily vapers over the 2020-2024 period became increasingly represented by youths from demographic groups that experience health disparities (females, non-Hispanic Black youths, and rural youths) and youths who used other substances.

The previously documented trend of intensifying youth vaping patterns from 2014 to 2021 seems to have persisted in recent years, albeit at a slower rate since 2022.^1^ Increasing daily nicotine vaping raises concerns, as daily vaping may be associated with more adverse cardiovascular, respiratory, addiction, and mental health outcomes compared with less frequent vaping.^1,5,21,22^ This study also provides new evidence that youths who are daily vapers may be hardening, given the finding of increasing unsuccessful quit attempts, which may have meaningful clinical implications (ie, implying treatment resistance or access barriers). To our knowledge, these results report the first recent estimates of cross-year trends in unsuccessful quitting in vaping US youths, demonstrating that more than half of daily vapers reported unsuccessful quitting in 2024. Prior similar national studies were limited to single-year point estimates of unsuccessful quitting prevalences of 11% in youths with lifetime vaping in 2020^23^ and 25% in youths who vaped 4 or more of the past 30 days in 2017.^24^ A history of unsuccessful quit attempts is a cardinal symptom of nicotine use disorder and may reflect the loss of control over nicotine use within the addiction process.^25^ In light of a recent clinical trial demonstrating strong efficacy of varenicline to treat nicotine dependence in young adults with frequent nicotine vaping,^26^ efforts to support clinician knowledge about the most efficacious treatments for nicotine dependence in youths with frequent nicotine vaping are needed.

Youths who vape may be hardening toward daily, relapsing use patterns for several reasons. These findings may be partially driven by a potential positive trend of increasing motivation to quit and of quitting attempts overall due to increasing perceived harms and social ostracization of vaping, which merits further study.^11,27,28^ Adolescents might also be gravitating toward using more addictive vaping products as the market shifts to products that are disposable, contain higher nicotine concentrations, have more puffs per unit, and contain nicotine salt formulations.^4,29,30,31^ Prior research shows that adolescents who vape disposable and higher nicotine concentration products (vs not) are at higher risk of persistent, frequent vaping.^29,30^ MTF collected data on nicotine potency of vaping products used by youths in 2024, showing that 48% of users vaped products with either very high or ultrahigh potency, with nicotine concentration of 5% or 6% or greater, respectively.^3^ Further study is needed to evaluate cross-year changes in nicotine concentration, shifts in nicotine products of daily users, and how use of those products may influence users’ ability to quit.

This study’s findings suggest that the youth vaping population might be hardening due to increasing representation by youths with behavioral health problems. Adolescents who used cannabis, alcohol, or tobacco products other than e-cigarettes also did not show the same substantial declines in current nicotine vaping prevalence over 2020 to 2024 compared with the nonusing population. Prior work shows substantial overlap between drivers of cannabis and nicotine vaping in youths (eg, psychoactive effects, social reasons, and coping with mental health symptoms) and associations between frequency of use of both substances.^32,33^ Furthermore, adverse effects of vaping nicotine or cannabis alone may be amplified in youths who use both substances, including neurocognitive impairments (ie, memory problems, mood disorders), psychiatric comorbidities, respiratory symptoms, and increased likelihood of persistent substance use into adulthood.^32,33,34,35,36^ Clinicians and prevention practitioners should be mindful that youths who vape are increasingly demonstrating co-use patterns, which pose treatment challenges and highlight important opportunities for early prevention efforts. While polysubstance use is a known negative predictor of quit intentions and attempts,^17,37^ we did not find an association between using substances other than e-cigarettes and unsuccessful quit attempts in our interaction analysis. Additionally, trends in vaping outcomes did not differ by depressive symptoms and conduct problems, indicating the overrepresentation of these behavioral and mental health issues in youths who vape does not appear to be changing over time.

The youth vaping population also appears to be shifting toward increasing representation by females and non-Hispanic Black youths. Sex differences identified herein align with findings of Mattingly and Hart,^38^ demonstrating an inversion of sex differences in the prior decade, whereby youth e-cigarette use prevalence was higher in males than females in 2015 but higher in females than males in 2021. The current study provides new evidence that this sex disparity in youth nicotine vaping may have further widened by 2024. Mattingly and Hart^38^ also found decreasing current vaping prevalence in all racial and ethnic groups from 2019 to 2020, which rebounded to increases from 2021 to 2022 for non-Hispanic Black and Hispanic youths only.^38^ While the current study did not show increases in nicotine vaping among any race and ethnicity group during the 2020-2024 period, non-Hispanic Black youths did not experience the same declining trend as the other race and ethnicity groups. Females have been shown to be more susceptible to the nonpharmacologic (sensory-taste) mechanisms that underlie nicotine addiction than males.^39^ Race and ethnicity differences in nicotine use could reflect disproportionate exposure to socioeconomic disadvantage, discrimination, industry marketing, easily accessible tobacco products, and tobacco control policies.^40,41,42,43,44^ Clinicians and public health professionals should be aware of these shifting sex and racial-ethnic distributions in youths who vape by ensuring universal screening for these populations and ensuring knowledge of evidence-based behavioral interventions that address nonpharmacologic and social determinants of nicotine use in these groups.

While recent studies have demonstrated shifting geographic variation in current nicotine vaping,^45^ this study’s novel focus on daily vaping outcomes revealed a substantial 2-fold increase between 2020 and 2024 for this outcome in rural youths. Potential drivers include shifts in social norms and harm perceptions of e-cigarettes compared with combustible cigarettes, targeted vaping marketing, decreased exposure to antitobacco or antivaping campaigns, experience of psychosocial stressors specific to rural communities, and gaps in prevention and cessation access in youths in rural communities.^46,47,48,49^ Taken together, further efforts are needed to increase prevention and cessation access to rural youths who vape nicotine and are at disparate risk for daily use.

### Limitations

Our study has limitations. Self-report measures are subject to recall error. We did not assess trends in overall quit attempts or successful quit attempts (ie, in former users), which may also be increasing.^23^ Due to COVID-19, data collection was either abbreviated or conducted remotely for some students in certain years but not others, which may have impacted trend estimates; however, several factors reduce the likelihood that these changes had substantial impacts. First, the abbreviated 2020 MTF sample was demographically similar to previous years, indicating no substantive differences in sampling bias across years.^28^ Second, remote data collection methods would be expected to produce underreporting due to confidentiality concerns, but the prevalence of daily vaping and unsuccessful quit attempts increased in 2021 when a majority of surveys were completed remotely.

## Conclusions

In this cross-sectional study of US youths, we found a trend of hardening among youths who vape nicotine, highlighting the need for expansion of evidence-based prevention and cessation programs, particularly for certain subpopulations identified as at disparate risk. Tailored cessation interventions for youths with frequent nicotine vaping, with particular focus on certain demographics (ie, females, non-Hispanic Black youths), on those who use cannabis, alcohol, or other tobacco products, and on those living in rural communities, may be warranted to offset the progression of these trends and worsening of related negative health outcomes.

## Notes

Supplement 1. **eTable.** Interaction of Respondent Characteristics × Year in Predicting Outcomes, Omnibus *P* Values **eFigure.** Study Accrual Flowchart

Supplement 2.
Data Sharing Statement
