# Supplementary material for: Trends in Daily Nicotine Vaping and Unsuccessful Quit Attempts in Youths
Source: JAMA Netw Open. 2025 Nov 3;8(11):e2541061. doi: 10.1001/jamanetworkopen.2025.41061 (PMC12584035; doi:10.1001/jamanetworkopen.2025.41061)
Supplement: Supplement 1. — eTable. Interaction of Respondent Characteristics × Year in Predicting Outcomes, Omnibus P Values eFigure. Study Accrual Flowchart [file jamanetwopen-e2541061-s001.pdf]

Supplementary Online Content

Masonbrink AR, Bae D, Cho J, et al. Trends in daily nicotine vaping and unsuccessful quit attempts in youths. *JAMA Netw Open*. 2025;8(11):e2541061. doi:10.1001/jamanetworkopen.2025.41061

eTable. Interaction of Respondent Characteristics × Year in Predicting Outcomes, Omnibus *P* Values

eFigure. Study Accrual Flowchart

This supplementary material has been provided by the authors to give readers additional information about their work.

© 2025 Masonbrink AR et al. *JAMA Network Open*.

eTable. Interaction of respondent characteristics x year in predicting outcomes, omnibus p-values[a]

Characteristics	Overall sample (analytic sample A) N=115,191<br>Outcome: Past-30-day use (yes vs. no)	Among past-30-day vapers (analytic sample B) N=15,226<br>Outcome: Past-30-day daily use (yes vs. no)	Among past-30-day daily vapers[a] (analytic sample C) N=3,512<br>Outcome: Unsuccessful Quit Attempt (yes vs. no)
Grade	.14	.13	.20
Female	<.001[f]	.71	.71
Race/ethnicity	.001[f]	.50	.13
Population density	.11	.001[f]	.51
Conduct problem (any vs. no)[b]	.43	.91	.99
Depressive symptom (above vs. below median)[c]	.23	.89	.06
Past 30-day Substance use			
Tobacco (yes v. no)[d]	<.001[f]	.11	.19
Cannabis (yes vs. no)[e]	<.001[f]	.003[f]	.34
Alcohol (yes vs. no)	.003[f]	.41	.89

[a]Daily vaping is defined as vaping on all 30 days during the past 30-day period.
[b]Conduct problems were assessed using five self-reported items on the frequency of specific behaviors in the past 12 months (Once–5+times[=any] vs. None; e.g., "hurt someone badly," "taken something not belonging to you worth over $50"). These items were administered to a randomly selected subsample, reducing the analytic sample to 25,045 for all MTF respondents, 2,855 for past-30-day vapers, and 587 for daily vapers.
[c]Depressive symptoms were assessed using four self-reported items (e.g., "Life often seems meaningless," "The future often seems hopeless"), summed to create a total score (range: 4–20). Participants scoring above the sample median were classified as having depressive symptoms (yes/no). These items were administered to a randomly selected subsample, reducing the analytic sample to 44,390 for all MTF respondents, 5,402 for past-30-day vapers, and 1,253 for daily vapers.
[d]Cigarette, smokeless tobacco, large cigars, flavored little cigars/cigarillos, regular little cigars/cigarillos, hookah tobacco.
[e]Smoking and vaping marijuana.
[f]Statistically significant after Benjamini-Hochberg correction for multiple tests to maintain study-wise false discovery rate=.05.

© 2025 Masonbrink AR et al. *JAMA Network Open*.

eFigure. Study accrual flowchart

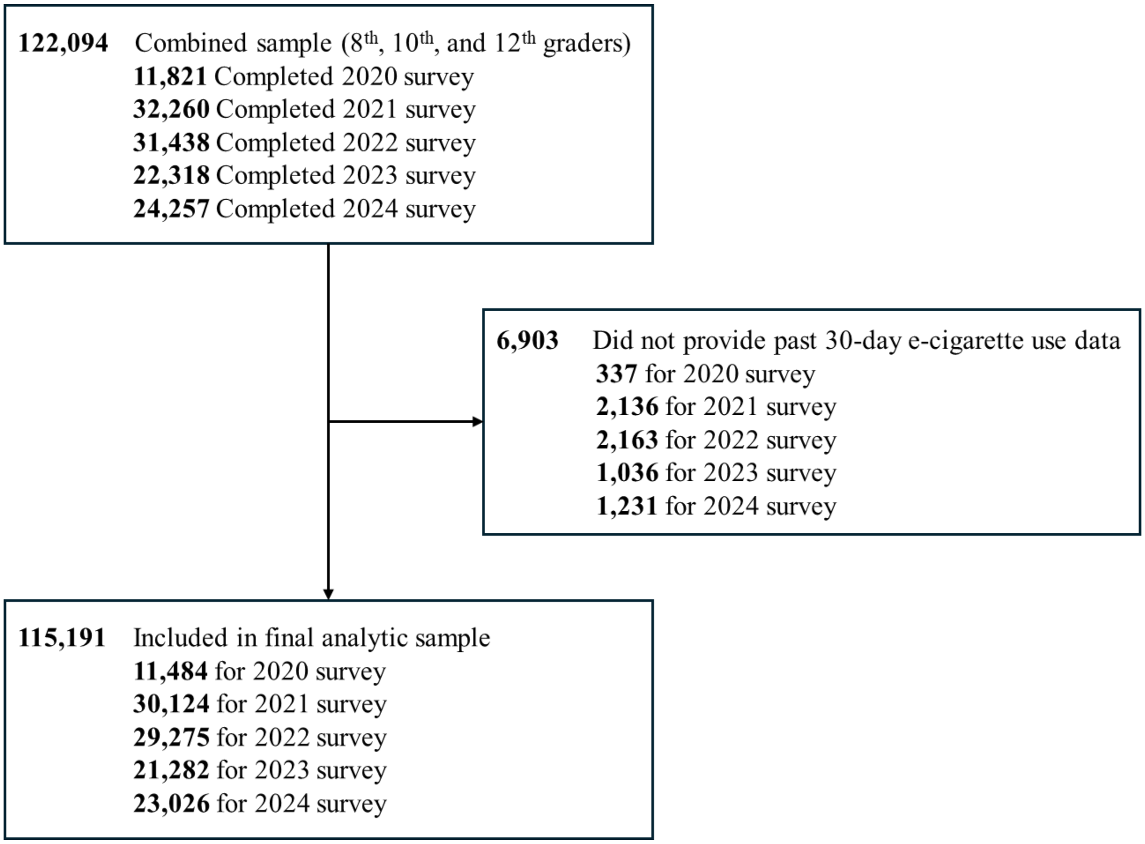

© 2025 Masonbrink AR et al. *JAMA Network Open*.
