# Supplementary material for: Trends in Daily Nicotine Vaping and Unsuccessful Quit Attempts in Youths
Source: JAMA Netw Open. 2025 Nov 3;8(11):e2541061. doi: 10.1001/jamanetworkopen.2025.41061 (PMC12584035; doi:10.1001/jamanetworkopen.2025.41061)
Supplement: Supplement 2. — Data Sharing Statement [file jamanetwopen-e2541061-s002.pdf]

Data Sharing Statement

Masonbrink. Trends in Daily Nicotine Vaping and Unsuccessful Quit Attempts in Youths. *JAMA Netw Open*. Published November 03, 2025. doi:10.1001/jamanetworkopen.2025.41061

Data
Data available: No
